# A rare combination of hypogonadotropic hypogonadism, GH deficiency and rectal atresia in a female with an FGFR1 variant: a case report and systematic review of the literature

**DOI:** 10.1007/s12020-025-04261-4

**Published:** 2025-05-28

**Authors:** Krystallenia I. Alexandraki, Odysseas Violetis, Eleni Memi, Helen Fryssira, Vasileios Papanikolaou, Maria Papagianni, George Mastorakos

**Affiliations:** 1 2nd Department of Surgery, Aretaieio Hospital, National and Kapodistrian University of Athens, Athens, Greece; 2 Unit of Endocrinology, Diabetes mellitus, and Metabolism, Aretaieion Hospital, School of Medicine, National and Kapodistrian University of Athens, Athens, Greece; 3 Laboratory of Medical Genetics, National and Kapodistrian University of Athens, Athens, Greece; 4 Department of Otorhinolaryngology, General Chest Hospital “Sotiria”, Athens, Greece; 5 Department of Nutrition and Dietetics, School of Physical Education, Sport Science and Dietetics, University of Thessaly, Trikala, Greece; 6 Endocrine Unit, 3rd Department of Pediatrics, Hippokration Hospital of Thessaloniki, Aristotle University of Thessaloniki, Thessaloniki, Greece

**Keywords:** FGFR1, CPHD, Hypopituitarism, GHD, Review

## Abstract

**Purpose:**

To report a case with combined pituitary hormone deficiency (CPHD) and Fibroblast growth factor receptor 1 (FGFR1) gene defect, and summarize the clinical characteristics of similar cases by reviewing the current reports from the literature.

**Methods:**

A 24-year-old woman was admitted to the outpatient endocrinology unit with a diagnosis of primary amenorrhea, history of Growth Hormone deficiency and multiple congenital anomalies including rectal atresia. The subsequent hormonal investigation led to the diagnosis of hypogonadotropic hypogonadism and persistent GH deficiency. Abdominal and pelvic ultrasounds were normal whereas the brain MRI revealed a hypoplastic sella turcica with a hypoplastic anterior pituitary lobe, an ectopic posterior pituitary lobe and a thin pituitary stalk. The genetic analysis revealed a novel pathogenic missense heterozygous variant (*c.1958G* *>* *A, p.Agr635Gln*) in exon 15 of FGFR1 gene. PubMed, Scopus, and Web of Science were searched for the identification of studies reporting cases of CPHD with FGFR1 gene defects.

**Results:**

Of the 648 records retrieved, 10 were included in this review. A comprehensive overview of the cases was summarized, and their clinical and genetic characteristics were presented.

**Conclusion:**

Although FGFR1 variants have been associated with Kallmann syndrome and isolated hypogonadotropic hypogonadism and recently with CPHD, the patient’s phenotype includes phenotypic alterations not previously described, to the best of our knowledge, within the spectrum of non-reproductive features of either of these entities. Isolated GH deficiency combined with other non-common abnormalities exerts a great possibility for subsequent CPHD manifestation.

## Introduction

Congenital hypogonadotropic hypogonadism results from genetically caused deficiency of gonadotropin releasing hormone (GnRH). It manifests with absent or incomplete puberty. Patients are often diagnosed at post-puberty even in early adulthood [1]. In females, primary amenorrhea and delayed breast development are the prominent signs of congenital hypogonadotropic hypogonadism in puberty, whereas secondary amenorrhea, fertility problems and osteoporosis are the main reasons leading an adult woman to seek medical advice [2]. Although until recently congenital hypogonadotropic hypogonadism (CHH) was believed to be an isolated disorder there is enough literature to support that it can be part of combined pituitary hormone deficiency (CPHD) which encompasses other pituitary hormone deficiencies as well as septo-optic dysplasia (SOD) [2, 3]. Diagnosis of SOD requires at least two of the following: 1) midline defects such as corpus callosum agenesis, 2) optic nerve hypoplasia, or 3) anterior pituitary hypoplasia and consequent endocrine deficiencies [4]. The development of pituitary gland requires a highly complex interplay of transcription factors and signaling pathways, interruption of which leads to both CHH and CPHD [5, 6].

Combined pituitary hormone deficiency encompasses deficits in genes which participate in early and late embryonic development of the hypothalamic-pituitary region, with the most prevalent being *POUF1, PROP1, HEXS1, SOX3, LHX3, LHX4* [7]. Congenital hypogonadotropic hypogonadism is genetically heterogeneous implicating numerous genes, which are associated with the embryonic differentiation and migration of GnRH neurons or their function. Kallmann syndrome is the form of CHH that combines defective sense of smell and is responsible for about half of the cases of CHH. The causative genes of Kallmann syndrome and normosmic idiopathic hypogonadotropic hypogonadism might be different or overlapping. Overall, they account approximately for 40% of CHH cases [8]. Oligogenicity, defined by a phenotype when more than one variant is implicated, contributes to the genetic complexity and is estimated that it occurs in roughly 20% of cases [9]. Mutated genes, such as anosmin-1 (KAL1), prokineticin 2 (PROK2), prokineticin receptor 2 (PROKR2), GnRH receptor, fibroblast growth factor (FGF) 8, FGF receptor (FGFR) 1 are often found in patients with CHH. Furthermore, cases of reversible hypogonadism have been described and a reversibility rate of 10% has been reported [10].

A case of a patient with hypogonadotropic hypogonadism and persistent growth hormone (GH) deficiency whose genetic analysis uncovered a pathogenic missense heterozygous variant in exon 15 of the *FGFR1* gene is presented. A systematic review of the literature regarding the prevalence of *FGFR1* gene defects in patients with hypopituitarism was conducted as well, to shed light on the different phenotypes associated with this genetic abnormality.

## Materials and methods

**Fig. 1:**
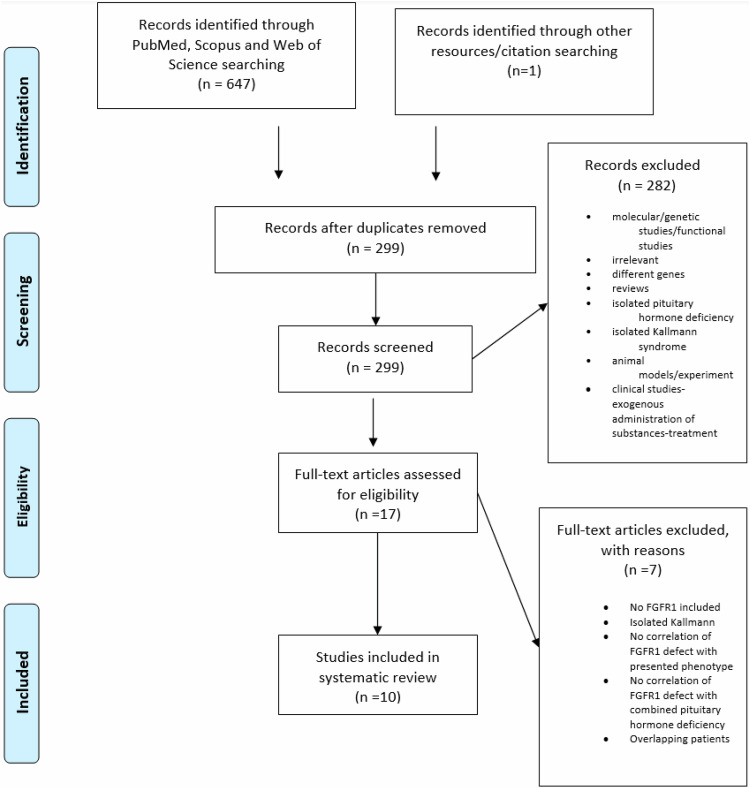
PRISMA Flow Diagram

Identification of FGFR1 variants in patients presenting with CPHD was targeted. A systematic review of the literature scrutinizing three databases, namely PubMed, Scopus, and Web of Science was conducted. This systematic review was performed following recommendations of the Preferred Reporting Items for Systematic Reviews and Meta-Analyses (PRISMA) [11]. Without limitations based on the type of study (case reports, case series, cohort studies, case-control, conference abstracts), patients presented with CPHD, hypopituitarism or Kallmann syndrome associated with other pituitary deficiencies presenting FGFR1 gene defects were included in the present review. The search strategy followed an extensive literature search using the following terms in the abstract or title areas: pituitary deficiency, hypopituitarism, Kallmann syndrome and FGFR1 up to November 2024. Boolean operators (AND, OR) were also used to narrow down the search. References of the upcoming relevant articles were also searched for identification of additional studies. Two independent reviewers (O.V., K.I.A) screened the titles and abstracts of the articles and selected potentially relevant studies (Fig. 1). Full-text articles were then studied, and the final articles were selected based on the inclusion and exclusion criteria. Disagreements were resolved through discussion and intervention of a third reviewer (G.M.) when needed. All studies were carefully compared to avoid the inclusion of duplicate or overlapping samples.

### Case report

A 24-year-old woman was admitted to a university outpatient endocrinology unit with a history of primary amenorrhea, for which she was on hormone replacement therapy (HRT) (combined pill with 2 mg estradiol valerate and 0.5 mg norgestrel) *per os* since the age of 18 years old. She had a history of surgically repaired rectal atresia, congenital dislocation of the right hip and ptosis of the left eyelid. In her medical data, she had vesicoureteral reflux with functionally impaired right kidney and GH deficiency, for which she had been treated with GH replacement therapy for several years during childhood. Recently, she had undergone orthopedic surgery owing to patella fracture. She described a normal sense of smell. In her family history, her father perished after he was diagnosed with acute myeloid leukemia, whereas the other family members, notably her mother and her two sisters are well and healthy. Her treatment, besides HRT, consisted of 100 mg allopurinol OD for hyperuricemia and calcium and vitamin D supplements for osteopenia. Her height, weight and body mass index were 160 cm, 80 kg and 31.25 kg/m2, respectively. She had normal breast (Tanner V) and pubic hair (Tanner V) development. She was presented with hypοtelorism, left epicanthus, low set-ears with pinna abnormalities and dental deformities.

While being in her regular treatment, laboratory tests taken fasting at 8.00 a.m. were as follows: free thyroxine (fT4) 0.78 ng/dL(0.71–1.48 ng/dL), thyroid stimulating hormone (TSH) 2.56 μIU/mL (0.4–4 μIU/mL), follicle-stimulating hormone (FSH) 0.9 mU/mL (3–8 mU/mL), luteinizing hormone (LH) < 0.07 mU/mL (2–7 mU/mL), prolactin (PRL): 16.6 ng/ml (4–24 ng/ml), estradiol (E2): 18 pg/ml (25–145 pg/ml), cortisol (8am): 16.4 μg/dl (6–19.5 μg/dl) and adrenocorticotropic hormone (ACTH): 42 pg/ml. After discontinuing all her medications for three months her laboratory tests were as follows: FSH 2.8 mU/mL (3–8 mU/mL), LH 1.2 mU/mL (2–7 mU/mL), PRL: 10.3 ng/ml (4–24 ng/ml), E2: 17 pg/ml (25–145 pg/ml), total testosterone: 0.29 ng/ml (0.1–0.6 ng/ml), dehydroepiandrosterone sulfate (DHEAS): 235 μg/dl (148–407 μg/dl), androstenedione: 244 ng/dl (120–400 ng/dl), 17-hydroxyprogesterone: 0.78 ng/ml (0.2–1.2 ng/ml) and sex hormone binding globulin (SHBG): 28.5 nmol/l (26–110 nmol/l) (Table 1a). The rest hematological, biochemical and urine tests were normal except for fasting glucose, lipid profile and uric acid which were above normal limits (Table 1b). Peak FSH and LH levels during LH-releasing hormone (LHRH) stimulation test were low (FSH: <0.73 mU/ml and LH: <0.5 mU/ml) suggesting hypogonadotropic hypogonadism. After performing 1 mg i.m glucagon test for GH evaluation, peak plasma GH concentration was <1 ng/ml, which is diagnostic for GH deficiency in obese patients [12, 13]. Insulin-like growth factor-I (IGF-1) was at lower normal levels (90 ng/ml N: 88–537 ng/ml). The ACTH-stimulation test (Synachthen test) was normal (Table 2).

**Table 1:** Hormonal and biochemical investigation of the patient during Hormone Replacent Treatment (HRT) and 3 months after HRT discontinuation

	HRT(+)	HRT(−)		HRT(+)	HRT(−)
**FSH (mU/ml)**	0.9	2.8	**PTH (pg/ml)**		16.8
**LH (mU/ml)**	<0.07	1.2	**Insulin (μU/ml)**		16.6
**E2 (pg/ml)**	18	17	**Cortisol 8PM (μg/dl)**	16.4	13.1
**PRL (ng/ml)**	16.6	10.3	**ACTH 8PM (pg/ml)**	42	39.3
**TSH (μU/ml)**	2.56	2.85	**SHBG (nmol/l)**		28.5
**FT4 (ng/dl)**	0.78	0.78	**Δ4A (ng/dl)**		244
**T3 (ng/dl)**	1.14		**17OHPG (ng/ml)**		0.78
**Anti-TPO (U/ml)**	<1		**Testosterone (ng/ml)**		0.29
**Anti-TG (U/ml)**	3		**DHEAS (μg/dl)**		235
**IGF1 (ng/ml)** (101–358)	137		**FAI**		3.5
**WBC** (x10^3^/uL)	9,22	7,00	**Urea (mg/dl)**	23,6	36,7
**HCT** %	38,6	39,0	**Creatinine (mg/dl)**	0,7	0,7
**HGB** (g/dl)	13,2	13,3	**Na**^**+**^ **(mmol/l)**	136	141
**PLT** (x10^3^/uL)	232	302	**K**^**+**^ **(mmol/l)**	4,1	4,7
**ΑLP** (U/L)		68	**Ca**^**++**^ **(mg/dl)**	10,2	10,4
**GGT** (U/L)		21	**Albumin(g/dl)**	4,7	4,5
**SGOT** (U/L)	13	16	**Ca**^++^ **corrected. (mg/dl)**	9,64	10
**SGPT** (U/L)	13	31	${\bf{P}}{{{\bf{O}}}_{{\bf{4}}}}^{{\bf{3}}-}({\bf{m}}{\bf{g}}/{\bf{d}}{\bf{l}})$		4,2
**LDH** (U/l)		161	**Mg**^**++**^ **(mg/dl)**		1,9
**Chol. (mg/dl)**	252,4	236,8	**Chol. (mg/dl)**	252,4	236,8
**TG (mg/dl)**	170	169,5	**TG (mg/dl)**	170	169,5
**HDL (mg/dl)**	41,5	44,7	**HDL (mg/dl)**	41,5	44,7

*WBC* white blood cells, *HCT* hematocrit, *HGB* hemoglobin, *PLT* platelets, *ALP* alkaline phosphatase, *GGT* gamma-glutamyl transpeptidase, *SGOT* aspartate transaminase, *SGPT* alanine transaminase, *LDH* lactate dehydrate, *Chol*. total cholesterol, *TG* triglycerides, *HDL* high-density lipoprotein, *LDL* low-density lipoprotein, *FSH* follicle-stimulating hormone, *LH* luteinizing hormone, *E2* estradiol, *PRL* prolactin, *TSH* thyroid-stimulating hormone, *anti-TPO* anti-thyroid peroxidase antibodies, *anti-TG* antithyroglobulin antibodies, *IGF1* insulin-like growth factor 1, *PTH* parathyroid hormone, *ACTH* adrenocorticotropic hormone, *SHBG* sex hormone binding globulin, *Δ4Α* androstenedione, *17OHPG* 17-Hydroxyprogesterone, *DHEAS* dehydroepiandrosterone sulfate, *FAI* free androgen index, *HRT (+)* on hormone replacement treatment, *HRT (-)* three months after discontinuation of Hormone Replacement Treatment

**Table 2:** Provocation tests of the patient 3 months after Hormone Replacement Treatment discontinuation

	Stimulus	0′	15′	30′	60′	90′	120′	150′	180′
FSH (mU/ml)	GnRH 100 μg i.v	<0.73	<0,73	<0.73	<0.73				
LH (mU/ml)		<0,5	<0,5	0,5	0,5				
Cortisol (μg/dl)	Synacthen 0.25 mg i.v	6,7		22,6	28,8				
ACTH (pg/ml)		30,7							
Glucose (mg/dl)	Glucagon 1 mg i.m	94		152	181	164	131	100	87
GH (ng/ml)		<0,1		<0,1	<0,1	0,4	0,7	0,2	0,1

*FSH* follicle-stimulating hormone, *LH* luteinizing hormone, *GnRH* gonadotropin-releasing hormone, *ACTH* adrenocorticotropic hormone, *GH* growth hormone

Abdominal and pelvic ultrasounds showed uterus with small dimensions and thin endometrium, ovaries without pathology and normal size (right ovary: 2.6 ×1.7 cm, left ovary: 2.7 ×1.3 cm), small sized right kidney (longitudinal axis: 5.8 cm) and parapelvic cysts in the left kidney (diameter: < 15 mm). Osteoporosis was confirmed after a bone density scan was performed [L2-L4 region with bone mass density (BMD): 0.900 g/cm2, Z-score: −3.2; left femur neck with BMD: 0.838 g/cm2, Z-score: −1.8]. Brain magnetic resonance imaging (MRI) revealed a hypoplastic sella turcica with a hypoplastic anterior pituitary lobe (5.5 × 4 × 3 mm), an ectopic posterior pituitary lobe and a thin pituitary stalk.

Both the patient and her mother underwent nasal endoscopy evaluation which revealed nasal septum deviation. Subsequently, olfactory sense was evaluated by Burghart Messtechnik kit which recorded score 11/12 in our patient and 12/12 in her mother, thereby hyposmia was ruled-out.

Karyotype was normal (46, XX). Genetic investigation with comprehensive whole exome next-generation sequencing revealed a pathogenic missense heterozygous variant (*c.1958G* *>* *A, p.Agr635Gln*) in exon 15 of the *FGFR* 1 gene. Approximately 37 Mb (214.405 exons) of the Consensus Coding Sequences (CCS) were enriched from fragmented genomic DNA by >340.000 probes designed against the human (Nextera Rapid Capture Exome, Illumina) and the generated library was sequenced on an Illumina NextSeq or HiSeq 4000 platform (Illumina). All exons and intron boundaries (+/− 20 bp) were analyzed. Relevant variants were in-house validated (GENDIA, Belgium). In the NGS panel only variants in disease genes relevant to the phenotype of the patient were reported. The WES analysis did not include variants in the mitochondrial genome, nor larger deletions or duplications encompassing intron-exon boundaries, nor repeat amplifications or methylation anomalies. Only variants in disease genes relevant to the phenotype of the patient were reported in the analysis. The list of genes (referred to as Mendeliome) comprised some 4000–4500 genes and was derived from the PubMed literature and gene / variant databases (eg OMIM, HGMD, ClinVar, LOVD, and other variant databases). In addition, copy numbers were assessed and no chromosomal disequilibrium was found. The variant found was not listed in the NHLBI Exome Variant Database nor in the GnomAD database. It was also not listed in the ClinVar database. In silico prediction: SIFT: deleterious, Mutation Taster: disease-causing. Due to its frequency, it was classified as a pathogenic variant according to the MutaDATABASE criteria. Notably, there were no other variants in the panel that could be involved or contribute to an oligogenic presentation. Interestingly, the patient’s mother was shown to carry the same pathogenic variant, while she was free of any related pathogenic condition which could be attributed to the huge genetic heterogeneity that was seen in the family because of its reduced penetrance and variable expressivity. The array of comparative genomic hybridization did not reveal any chromosomal abnormality in the patient.

The patient was changed to HRT with transdermal estradiol hemihydrate, 100 μg twice a week and oral micronized progesterone, 200 mg in the morning and 200 mg in the evening daily between the 16th and the 25th day of the menstrual cycle [14]. She continued calcium carbonate 600 mg once daily (OD), cholecalciferol 400IU OD and allopurinol 100 mg OD. She was also prescribed denosumab, 60 mg once every 6 months, for osteoporosis [15]. No adverse effects were noted, and the treatment was well tolerated by the patient. Appropriate permission from the patient was obtained to present her case.

### Outcomes of the systematic review

The search strategy yielded 648 candidate studies, out of which 631 were excluded (duplicates, reviews, title or abstract irrelevant to the inclusion criteria). After full-text review, only 10 were consistent with the purpose of our systematic review and finally included (Fig. 1). The characteristics of the included studies are shown in Table 3.

**Table 3:** FGFR1 variants in Combined Pituitary Hormone Deficiency (CPHD) patients

STUDY	GENE VARIANTS	SEX	AGE (years)	DEFICIENCIES	OTHER FEATURES	MRI	FUNCTIONAL ANALYSIS
Vermeulen [18]	Microdeletion in FGFR1 gene (8p11.2)	M	20	GH, FSH, LH	Congenital spherocytosis, minor facial deformities, crumpled ears, micrognathia, anosmia	–	
Fukami [19]	Heterozygous deletion in FGFR1 gene (8.5 Mb 8p11.1-p 12, all exons)	F	20	GH normal at baseline-low post-stimulated, FSH, LH low at baseline and post-stimulated, fT4 low with normal TSH	Learning disability, epilepsy	Chiari type 1 malformation, syringomyelia	
	Missense p.V102I (c.304 G > A)	F					Low
	Missense p.S107L (c.320 C > T)	F					NS
Correa [20]	Missense p.Ser107Leu (c.320 C > T)	F	15	GH, FSH, LH		APH, EPP	NS
	Missense p.Arg448Trp (c.1342 C > T)	F	4	GH, FSH, LH, TSH		APH, EPP, absent stalk	Low
	Missense p.Pro772Ser (c.2314 C > T)	M	16	GH, FSH, LH, TSH, ACTH	micropenis	APH, EPP, thin stalk	NS
	Missense p.Pro772Ser (c.2314 C > T)	M	15	GH, FSH, LH, TSH	micropenis	APH, EPP, absent stalk	NS
Lyra [21]	Missense p.Ala36Pro (c.106 G > C)	M	1.2	GH, TSH, ACTH	Micropenis, patent foramen ovale	APH, EPP, absent stalk	
Erbaş [22]	Nonsense p. R622X (c. 1864 C > T)	M		GH, FSH, LH	micropenis, cryptorchidism, flat nasal root	normal	–
Vishnopolska [23]	Missense p. Tyr807His (c. 2419 T > C)	F	0,5	GH, TSH, ACTH	Depressed nasal bridge, frontal bossing, midfacial hypoplasia, neonatal jaundice, SGA	APH	
Raivio [24]	Missense p.Ser450Phe (c. 1349 C > T)	M		FSH, LH, AVP	Central incisor, ASD, VSD, brachydactyly, bradychycephaly, preauricular skin tags	Corpus callosum dysgenesis with thinning of the genu body and absence of the rostrum, septum pellucidum agenesis	Low
	Missense p. Pro483Ser (c. 1447 C > T)	F		GH, FSH, LH, TSH, ACTH	Cleft lip/palate, micropthalmia, coloboma, moderate learning disability	APH, EPP	Low
	Missense p. Thr112Thr (c. 336 C > T)	M		GH, FSH, LH	Abnormal eyes, seizures	Corpus callosum agenesis	–
Tornese [25]	Missense (c.976 C → G)	M	0.9	GH	anosmia	Olfactory bulbs hypoplasia.	
Sano [26]	Missense (c.176 A > T, p.(Asp59Val)	M	18	GH, TSH, FSH, LH,		APH, EPP	
Cavallaro [27]	Missense (c.2002G>A, p.Glu668Lys)	M	14	GH, LH/FSH	Cleft lip and palate, cryptorchidism	Hypoplastic olfactory sulci, caudal dislocation of the fronto-basal cortical gyri, impaired cranio-caudal development of the nasal pits, nasal septal deviation, defect of the alveolar process of the maxillary bone, maxillary and frontal sinuses hypoplasia and one palatalized supernumerary tooth.	
Present case	**Missense** **(p. Agr635Gln c.1958 G** > **A)**	F	24	GH, FSH, LH	Rectal atresia, congenital dislocation of the hip, eyelid ptosis, right vesicoureteral reflux, kidney cysts, hypertelorism, left epicanthus eyefold, low set-ears with pinna abnormalities and dental deformities	APH, EPP, thin stalk	–

*GH* Growth Hormone, *FSH* Follicle Stimulating Hormone, *LH* Luteinizing Hormone, *fT4* Free Thyroxine, *TSH* Thyroid-Stimulating Hormone, *ACTH* Adrenocorticotropic-Hormone, *AVP* Arginine-Vasopressin, *ASD* Atrial Septal Defect, *VSD* Ventricle Septal Defect, *APH* Anterior Pituitary Hypoplasia, *EPP* Ectopic Posterior Pituitary, *NS* No Significance, *SGA* Small-for-Gestational-Age

## Discussion

The present case-index patient had CPHD and the genetic analysis revealed a *FGFR1* missense variant (*p. Arg653Gln, c. 1958G* > *A*). Fibroblast growth factor plays an important role in cell proliferation and differentiation in several tissues. The FGFR belongs to the receptor tyrosine kinase family and has four isoforms (FGFR1-4). FGFR1 dimerizes and activates mitogen-activated protein (MAP) kinase and Phospholipase C (PLC) signaling pathways controlling thus, cell function [16]. The *FGFR1* gene expression appears to be essential in the development of olfactory bulbs, the number of GnRH neurons and their migration from nasal placode [17].

Although variants of *FGFR1* gene have been postulated to be classically associated with Kallmann syndrome and Normosmic Idiopathic Hypogonadotropic Hypogonadism, more recent studies advocate that they might be also involved in the formation of the midline brain structures such as the pituitary, optic region, and septum pellucidum causing a wider phenotype of CPHD and SOD. Apparently, the first detection of CPHD phenotype in a patient with FGFR1 variant was made by Vermeulen et al. 2002 who discovered a microdeletion in 8p.11.2 region of the *FGFR1* gene in a patient with congenital spherocytosis, minor facial deformities, crumpled ears, micrognathia, anosmia, GHD and hypogonadotropic hypogonadism [18]. In a cohort study of 69 Japanese patients with CPHD, a microdeletion was found in a woman presenting GHD, hypogonadotropic hypogonadism, central hypothyroidism, epilepsy, learning disability and abnormal brain MRI. In the same study two missense variants [*p.V102I (c.304* *G* > *A), p.S107L (c.320* *C* > *T)*] were described, but they were suggested to be benign polymorphisms [19]. In another cohort of 156 Brazilian patients with CPHD, three missense *FGFR1* variants were found in four unrelated patients presenting with anterior pituitary hypoplasia, ectopic posterior pituitary, pituitary hormone deficiencies varying from GHD to hypogonadotropic hypogonadism or panhypopituitarism. From the three variants found, two were considered as polymorphisms [*p.Ser107Leu (c.320* *C* > *T), p.Pro772Ser (c.2314* *C* > *T)*], whereas the third one (*p.Arg448Trp (c.1342* *C* > *T)*) was considered deleterious [20]. *FGFR1(c.106* *G* > *C)* missense variant was also detected in a male with ectopic posterior pituitary and pituitary stalk interruption syndrome presenting with CPHD, though probably not responsible for the phenotype [21]. Additional association of *FGFR1* and CPHD was reported in a case report describing a 16-year-old male with micropenis, undescended testes, flat nasal root in conjunction with GHD and hypogonadotropic hypogonadism but normal MRI and normal smell test. The genetic analysis revealed an heterozygous nonsense variant in *FGFR1 [p.Arg622X (c.1864 C* > *T)]* [22]. In a cohort study from Argentina, screening of 170 pediatric patients with CPHD or isolated GHD, investigated the presence of candidate genes responsible for these conditions. A novel pathogenic missense variant (*p.Tyr807His (c. 2419* *T* > *C)*) was discovered to a small-for-gestational age neonate deficient to GH, TSH and ACTH with midfacial hypoplasia, frontal bossing and depressed nasal bridge [23]. Furthermore, a study which placed emphasis on the genetic overlap of Kallmann syndrome, CPHD and SOD, examined 35 patients with CPHD and 68 patients with SOD and documented 3 heterozygous variants [*p.Ser450Phe (c. 1349* *C* > *T), p. Pro483Ser (c. 1447* *C* > *T), p. Thr112Thr (c. 336* *C* > *T*)] in 3 patients with SOD [24]. Interestingly, a combination of Kallmann syndrome and GHD was detected in 4-year-old male, who was first evaluated for decreased growth velocity and GHD. An MRI scan showed hypoplastic olfactory bulbs. Since his father was diagnosed with Kallmann syndrome, genetic analysis was performed showing a heterozygous variant in the *FGFR1* gene (*c.976* *C* → *G*), both in the patient and his father [25]. Similarly, a 7-year-old male was referred for endocrinological evaluation because of short stature and was ultimately diagnosed with CPHD. Anterior pituitary hypoplasia and ectopic posterior pituitary were seen in the MRI scan. Genetic analysis detected 2 heterozygous missense variants in *FGFR1 [c.176* *A* > *T, p.(Asp59Val))* and *KISS1R (c.769* *G* > *C, p.(Val257Leu)]*, respectively and the in silico studies confirmed only the pathogenicity of the FGFR1 gene. Collectively, it was postulated that the presented phenotype can be mainly attributed to the FGFR1 variant [26]. Another case report described a 14-year-old patient presented with short stature and pubertal delay. Endocrinological evaluation documented GHD and hypogonadotropic hypogonadism. The subsequent MRI scan showed normal pituitary gland, but hypoplastic olfactory sulci, though the patient’s smell was not impaired. Eventually, heterozygous missense variant *c.2002G* *>* *A* in *FGFR1* gene emerged by genetic analysis, which is deemed pathogenic [27]. An additional male patient with hypogonadotropic hypogonadism, was not included in the present systematic review since he did not present initially multi-hormone deficiencies but a heterozygous *c. 570* *G* > *A FGFR1* variant was documented. However, later in his life, he presented with macroprolactinoma causing pituitary apoplexy and subsequent hypopituitarism raising questions about the variant's role in pituitary tumorigenesis [28]. In addition, it was decided not to be included in this systematic analysis a likely pathogenic variant of FGFR1 [missense variant *(c.1591* *G* > *A, p.Glu531Lys*)] in a patient with Kallmann syndrome, diabetes insipidus and holoprosencephaly because it is believed that diabetes insipidus may be related to holoprosencephaly. The FGFR1 seems to be responsible for the co-presence of Kallmann syndrome and holoprosencephaly [29]

Phenotypically, affected individuals with Kallmann syndrome combined with *FGFR1* variants are enriched with non-reproductive signs, most prevalent of which are skeletal anomalies of the hand or feet, dental agenesis and midline defects, namely cleft lip/palate [9]. In the present case report, the index patient presented signs, such as rectal atresia, congenital dislocation of the hip, vesicoureteral reflux, which do not belong to the spectrum of non-reproductive features neither of Kallmann syndrome nor of CPHD/SOD pathologic entities. Although her mother was also shown to carry the same pathogenic variant, she was asymptomatic, in contrast to her daughter, which may be attributed to genetic heterogeneity.

Despite the fact that the majority of pediatric patients diagnosed with GHD have isolated GHD in their initial presentation, they can develop additional pituitary hormone deficiencies during a life-long follow-up. Because GHD may be a part of CPHD, its initial diagnostic work-up includes functional assessment of pituitary gland globally and MRI, in order to evaluate the hypothalamus-pituitary anatomy [30]. Research focuses on identification of risk factors for progression from isolated GHD to CPHD. Children with GHD of organic etiology such as intracranial tumors, history of brain surgery or radiotherapy are more likely to develop CPHD than those with idiopathic GHD. Hypothalamic-pituitary anatomical anomalies, such as ectopic posterior pituitary, anterior pituitary hypoplasia, pituitary stalk agenesis and midline brain defects, which can be detected on MRI, are indicative of further progression to additional anterior pituitary hormone deficiencies in patients diagnosed with GHD. Genetic analysis is generally recommended, since it might reveal genetic defects in genes responsible for the hypothalamic-pituitary development and function, which can be related to variable phenotypes including the development of CPHD [31].

## Conclusion

In conclusion, endocrinologists should bear in mind that *FGFR1* variants contribute to different phenotypes of CPHD. If additional genetic loci or environmental factors play a role in the expression of a certain phenotype, is to be discovered. Under no circumstances does the diagnosis of isolated GHD excludes other pituitary hormone deficiencies and a life-long monitoring is crucial in these patients. The discovery of novel genes will help clinicians to predict the risk these patients run for progression to additional pathologic entities.

## Data Availability

No datasets were generated or analysed during the current study.
